# Developmental changes in social attention and oxytocin levels in infants and children

**DOI:** 10.1038/s41598-017-02368-x

**Published:** 2017-05-31

**Authors:** Minaho Nishizato, Takashi X. Fujisawa, Hirotaka Kosaka, Akemi Tomoda

**Affiliations:** 1 Division of Developmental Higher Brain Functions, United Graduate School of Child Development, University of Fukui, Fukui, 23-3 Matsuoka-Shimoaizuki, Eiheiji-cho Fukui 910-1193 Japan; 2 Research Center for Child Mental Development, University of Fukui, 23-3 Matsuoka-Shimoaizuki, Eiheiji-cho Fukui, 910-1193 Japan

## Abstract

Oxytocin (OT) signalling represents one of the most critical systems involved in human social behaviour. Although several studies have examined the relationship between social functioning and peripheral OT levels, the association between OT and the development of social attention has not been well studied. Therefore, we investigated the developmental relationship between gaze fixation for social cues and OT levels during young childhood. We examined visual attention using an eye tracking system in infants and children (5–90 months of age) and measured the concentration of OT in saliva samples. We observed a negative association between age and both attention toward social cues and salivary OT levels, and a positive association between age and attention for non-social cues. We also observed that salivary OT levels were modulated by polymorphisms in oxytocin receptor (*OXTR)* rs53576. Our results suggest that there is an age-dependent association between visual attention for social cues and OT levels in infants and children, and that the development of visual attention to the eyes as social cues is associated with both *OXTR* polymorphisms and OT levels. Such findings indicate that OT and *OXTR* status may provide insight into the atypical development of social attention in infants and young children.

## Introduction

Oxytocin (OT), a neuropeptide secreted from the posterior pituitary, has physiological functions in labour and lactation, and increasing evidence indicates that OT plays an important role in modulating social behaviour in diverse species^1^. In humans, much research has suggested that OT facilitates the ability to infer the mental state of others from viewing the eye region^2, 3^ and can even selectively enhance the memory encoding of faces^4^. OT also modulates trust and generosity in interpersonal relationships^5, 6^. In fact, OT affects the activation of brain areas responsible for emotion, mentalisation, and cognitive control, including the amygdala and prefrontal cortex^5, 7^. In addition, research has suggested that oxytocin receptors (OXTRs) are expressed within specific brain areas such as the prefrontal cortex, cingulate cortex, and amygdala in rodents and sheep^8^, as well as in humans^9^. These areas are involved in social behaviour, including reproductive and maternal behaviours, affiliation and attachment, and reactivity to social stress in nonhuman mammals^10^. In humans, these brain areas are also referred to as “social-brain networks”^11, 12^. Thus, OT and *OXTR*, which comprise the oxytocinergic system, play crucial roles in human social behaviour.

Genetic polymorphisms that modulate oxytocin neurotransmission have been linked to individual differences in social behavior^13^. Specifically, the *OXTR* gene is considered to play an important role in regulating social behaviour by modulating the release of oxytocin^14^. A common, naturally-occurring single-nucleotide polymorphism (SNP)—*OXTR* rs53576—has been observed in an intron of the *OXTR* gene in humans.When presented with social stimuli (emotional faces), adults with the AA genotype exhibit decreased amygdala activation and increases in the functional correlation between the hypothalamus and amygdala compared with adults with the AG or GG genotypes^15^. At the behavioural level, a significant correlation between affective mutuality in parent–child interactions and empathy for children was observed in children with the GG genotype, but not for children with an A allele^16^. According to a recent meta-analysis, there are positive associations between the rs53576 polymorphism and general sociality: G allele homozygotes had higher general sociality than the A allele carriers^17^. Collectively, the literature suggests that the *OXTR* rs53576 polymorphism is associated with systematic neural and behavioural differences in social functioning.

The duration of eye gazing is predictive of one’s ability to interpret the intentions of others and the meaning of social situations^18^. Developmental research has also suggested that gaze fixation plays a key role in social development^19, 20^. Eye-tracking technology has several advantages for investigating visual attention to social cues in typical or atypical development during infancy and childhood^21^. Several studies have used eye tracking software to compare atypical responses for social cues (e.g., human upper body, geometric patterns, or social images, etc.), such as those that occur in autism spectrum disorders (ASD), to those of individuals who have undergone typical development^22, 23^. This approach enables researchers to measure, with high precision and accuracy, at what a participant is looking and for how long. Moreover, it offers an optimal balance between ecological validity and methodological constraints^24^. Eye tracking is therefore a valuable method for the detection and characterisation of subtle variations in patterns of visual attention to social cues. Moreover, eye-tracking technology is applicable to all populations from infants to adults, irrespective of their level of non-verbal and verbal ability^24^. Therefore, the different aspects of visual attention for social cues can be investigated similarly across various participant statuses, such as age, gender, and clinical condition.

The aim of the present study was to investigate the relationship between visual attention for social cues and salivary OT levels in infants and children, as well as the potential modulation of these factors by the *OXTR* rs53576 gene polymorphism. Although several studies have utilised eye-tracking to examine the relationship between social functioning and peripheral OT levels, or that between social dysfunction and the pattern of visual attention, few studies have investigated the associations between the two during typical development. Previously, we revealed that aging in preschool children has a considerable effect on visual attention toward social cues^25^. However, developmental changes in this process occurring during infancy and young childhood remain largely unexplored, and the interaction between developmental changes in OT concentration and visual attention remains unclear. Therefore, in the present study, we investigated the relationships among these factors by measuring patterns of visual attention for social cues and salivary OT levels using an eye-tracking system and an enzyme-linked immunoabsorbant assay, respectively. Further, we identified *OXTR* polymorphisms using SNP genotyping analysis. We hypothesized that low visual attention for social cues would be associated with low salivary OT levels and/or the *OXTR* “risk” allele, and that the associations between these factors would be modulated by developmental changes.

## Results

### Developmental changes in visual attention for social cues

The mean percentage of time spent fixated on each category of social cues is presented in Table 1, together with the standard deviation. We first determined whether there were any sex differences in fixation duration using separate independent *t*-tests for each variable. First, for “human face” stimuli with or without mouth motion, no significant sex differences were observed in the percentage of fixation time for either area of interest (AOI) [eye area without mouth motion: *t* (149) = −1.11, *p* = 0.26; with mouth motion: *t* (149) = 0.43; and the mouth area without mouth motion: *t* (149) = −0.49, *p* = 0.62; with mouth motion: *t* (149) = −0.23, *p* = 0.82]. In contrast, for the “people and geometry” stimuli, a significant sex difference was observed in the percentage of fixation time for both AOIs [people stimuli: *t* (147) = −2.70, *p* = 0.008; and geometry stimuli: *t* (147) = 2.15, *p* = 0.03], indicating that young girls were more attentive to people moving and less attentive to geometric shapes than young boys. For bodily motion stimuli, no significant sex difference was observed [Upright: *t* (149) = −1.79, *p* = 0.08; Inverted: *t* (149) = 0.93, *p* = 0.35.]. Finally, for “finger pointing” stimuli, a significant sex difference was observed in the percentage of fixation time spent on pointed-at objects [*t* (147) = −2.07, *p* = 0.04], indicating that young girls were more attentive to pointed-at objects than young boys, although no sex difference in attention for non-pointed-at objects was observed between groups [*t* (147) = 1.07, *p* = 0.28.].

**Table 1:** Mean values and coefficients of salivary OT levels and gaze fixation parameters.

		Total (n = 149)		Boys (n = 76)		Girls (n = 73)		*t*	Correlation	
		*M*	*SD*	*M*	*SD*	*M*	*SD*		Age	OT levels
Human Face (without mouth motion)	%Eyes	68.61	19.18	66.89	20.26	70.39	17.94	−1.11	−0.421***	0.238**
	%Mouth	16.58	14.91	15.99	14.92	17.19	14.98	−0.49	0.531***	−0.273**
	%Out of AOI	14.81	13.10	17.12	15.15	12.42	10.11	2.24*	0.011	−0.038
Human Face (with mouth motion)	%Eyes	25.35	18.27	25.98	17.58	24.69	19.05	0.43	−0.198*	0.243**
	%Mouth	58.25	21.85	57.83	21.52	58.68	22.18	−0.23	0.287***	−0.260**
	%Out of AOI	16.40	12.91	16.18	12.60	16.63	13.31	−0.21	−0.205*	0.097
People & Geometry	%People	52.12	15.37	48.86	16.07	55.51	13.92	−2.7**	−0.417***	−0.099
	%Geometry	27.16	15.99	29.90	16.78	24.32	14.71	2.15*	0.517***	0.056
	%Out of AOI	20.71	7.67	21.24	8.53	20.16	6.67	0.86	−0.244**	0.082
Biological Motion	%Upright	56.56	15.42	54.36	14.68	58.84	15.94	−1.79	0.096	−0.124
	%Inverted	34.26	14.54	35.35	13.66	33.12	15.42	0.93	−0.006	0.143
	%Out of AOI	9.18	10.46	10.29	12.62	8.03	7.50	1.33	−0.133	−0.017
Finger pointing	%Pointed	49.33	10.86	47.54	10.46	51.18	11.02	−2.07*	−0.395**	0.080
	%Non−pointed	9.49	7.41	10.12	7.64	8.82	7.15	1.07	0.526**	−0.186*
	%Out of AOI	41.19	10.18	42.33	10.18	40.00	10.11	1.41	0.039	0.059
Oxytocin levels (pg/ml)		73.26	63.43	68.94	55.97	77.76	70.47	−0.85	−0.438***	—

*p < 0.05, ** *p* < 0.01, ***p < 0.001.

Abbreviations: AOI, area of interest; OT, oxytocin.

**Figure 1:**
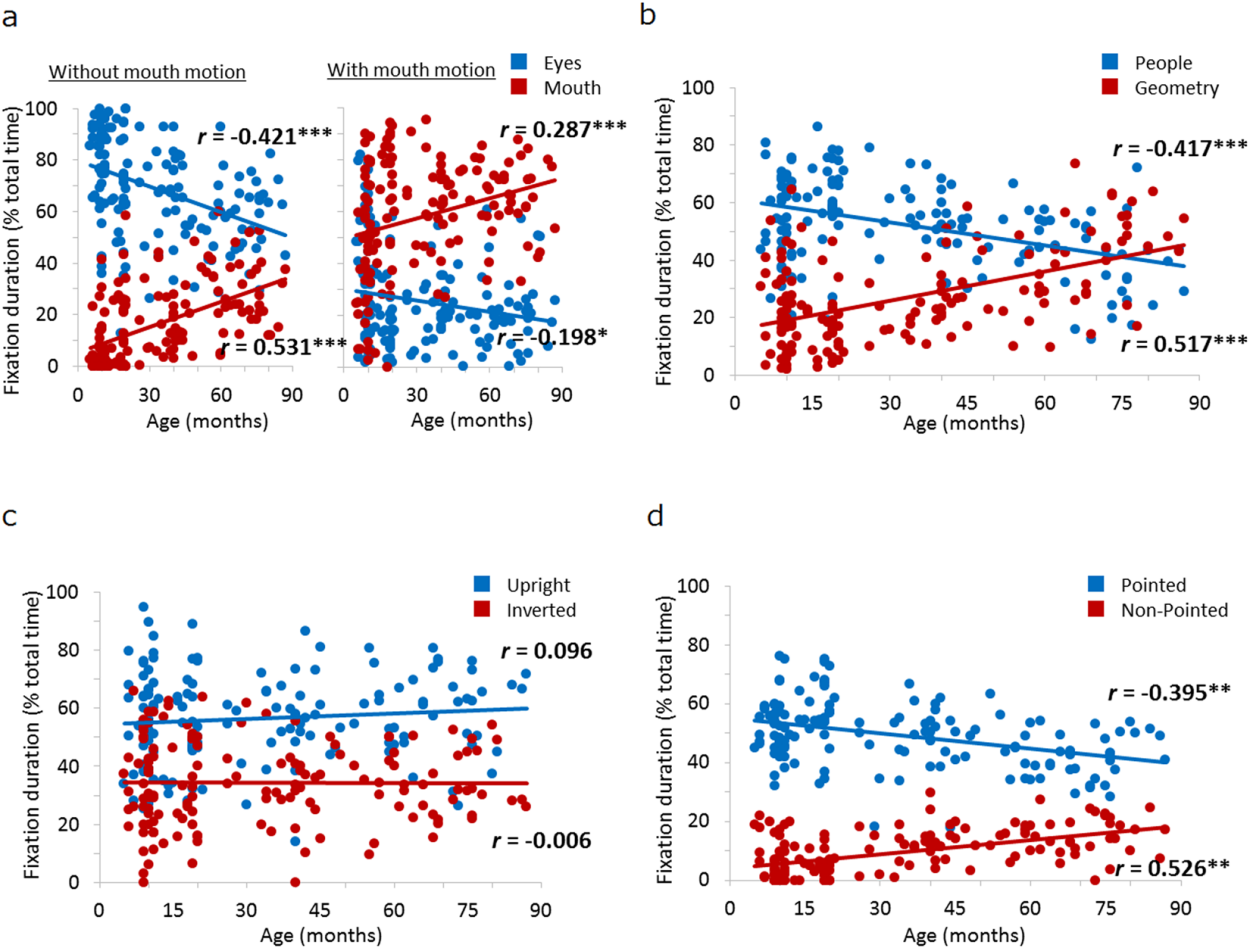
Correlations between age and percentage of the total time that eyes were fixated on social cues. (**a**–**d**). The vertical axis indicates fixation duration on each type of social stimulus as a percentage of total time measured. The horizontal axis indicates the age of the children in months. **p < 0.01; ***p < 0.001.

Next, we investigated the influence of age on fixation time for each category in order to address whether the pattern of attention to social cues is dependent on age. The correlation coefficients between age and each variable of fixation duration are presented in Table 1. Significant negative correlations were observed between age and fixation time for most AOI-1s (i.e., the higher social cues, which included the eye area of the “human face” category, people moving in the “people and geometry” category, and pointed-at objects in the “finger-pointing” category), with the exception of the upright figure in the “biological motion” category (Fig. 1). In addition, significant positive correlations were observed between age and fixation time for most AOI-2s (i.e., the non-social or lower social control cues, which included the mouth area in the “human face” category, geometric patterns in the “people and geometry” category, and non-pointed-at objects in the “finger pointing” category), again with the exception of the inverted figure in the “biological motion” category. These results suggest that visual attention for higher social cues decreases with age, whereas attention for non-social or lower social cues increases with age. This may be associated with increased behaviours related to exploration of the external environments and/or investigation of the associations between social cues and other non-social objects (e.g., joint attention).

### Developmental changes in salivary oxytocin levels

**Figure 2:**
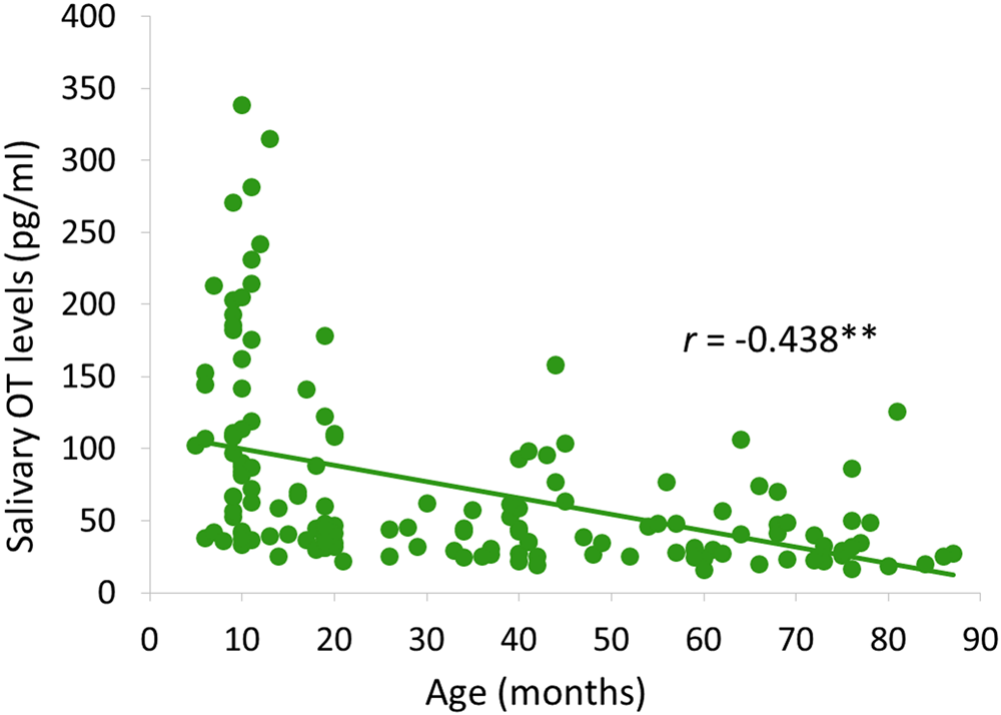
Relationship between age and salivary oxytocin (OT) levels. The vertical axis indicates salivary OT levels (pg/ml), whereas the horizontal axis indicates age. **p < 0.01.

Mean values of salivary OT levels are also presented in Table 1. No significant sex difference was observed with regard to salivary OT levels [*t* (149) = 0.85, *p* = 0.44]. We then investigated the potential influence of age on salivary OT levels (Table 1). A significant negative correlation was observed between salivary OT levels and age, suggesting that salivary OT levels decrease with age (Fig. 2).

In a subset of participants, we further examined whether OT levels are affected by age-associated fluctuations in levels of total protein (TP). We observed a significant negative association between age and salivary OT levels normalised by salivary TP levels, suggesting that salivary OT levels decrease with age regardless of TP levels (Supplementary Information).

### Relationship between gaze fixation duration and salivary OT levels

Because we observed parallel decreases in attention for social cues and salivary OT levels with age, we then examined the direct associations among these variables. As shown in Table 1, most of the significant correlations between gaze fixation duration and salivary OT levels were noted in the “human face” category. Positive correlations were observed between OT levels and the fixation time spent on the eye area of the face [without mouth motion: *r* = 0.238, *p* = 0.003; with mouth motion: *r* = 0.243, *p* = 0.003], whereas negative correlations were observed between OT levels and the fixation time spent on the mouth area [without mouth motion: *r* = −0.273, *p* = 0.001; with mouth motion: *r* = −0.260, *p* = 0.001]. These results suggest that children with higher OT levels were more attentive to the eye region in human faces than children with lower OT levels. Next, because both of these factors were influenced by age, we calculated partial correlation coefficients between salivary OT levels and the fixation times spent on each AOI in the face, controlling for age. Results indicated a weak yet significant positive partial correlation with a small effect size^26^ between OT levels and fixation time on the eye area in the “human face with mouth motion” category (*r* = 0.176, *p* = 0.03), indicating that children with higher OT levels were more attentive to the eye area of the face (with mouth motion) than children with lower OT levels, regardless of their age.

**Figure 3:**
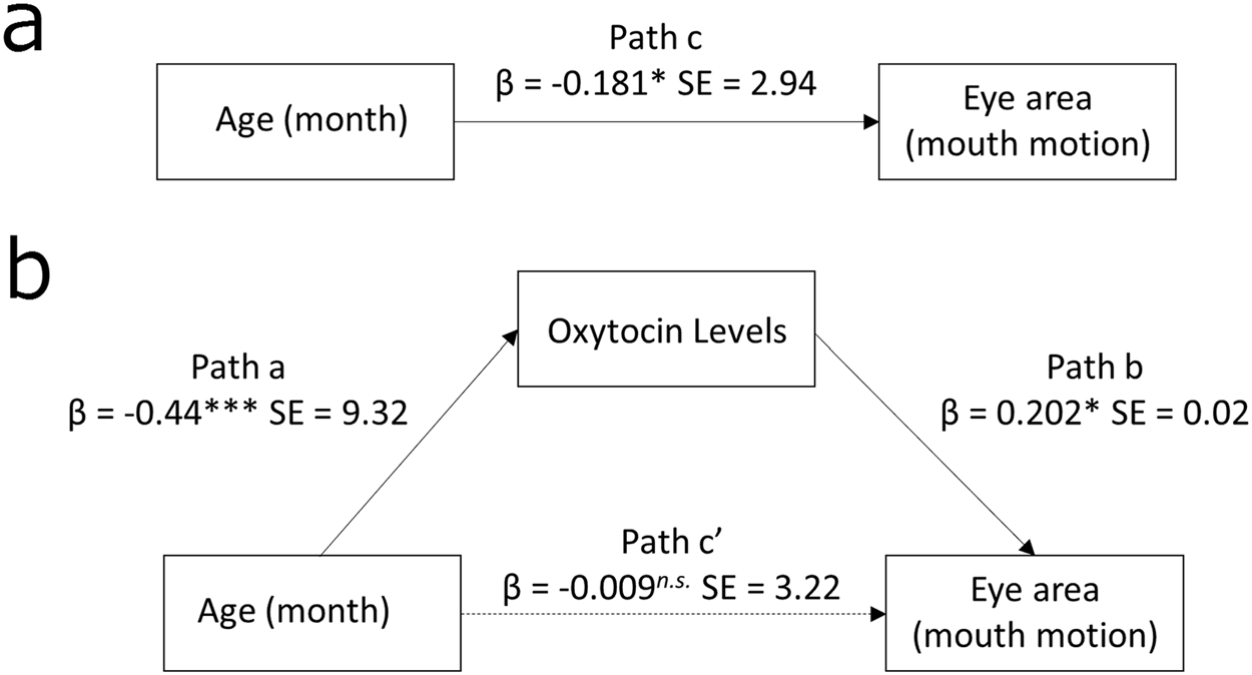
The role of oxytocin in mediating the influence of infant age on gaze fixation on the eye area (Human face [without mouth motion]). *p < 0.05; ***p < 0.001.

We then conducted a path analysis to examine whether OT levels mediate the relationship between age and visual attention to the eyes (Fig. 3). As shown in Fig. 3a, we observed that age was a significant predictor of gaze fixation on the eye area for infants and children in the present study (human face [without mouth motion]) (*β* = −0.181, *t* = 2.24, *p* = 0.025). As shown in Fig. 3b, age was also a significant predictor of oxytocin levels (*β* = −0.44, *t* = 5.99, *p* < 0.001). Furthermore, when oxytocin levels and age were entered simultaneously as predictors of fixation on the eye area (human face [without mouth motion]), oxytocin levels remained a significant predictor (*β* = 0.202, *t* = 2.29, *p* = 0.022), while age did not (*β* = 0.009, *t* = 1.03, *p* = 0.30). Thus, these findings suggest that oxytocin levels mediate the relationship between age and visual attention to the eye area (Human face [without mouth motion]).

### *OXTR* rs53576 polymorphism and salivary OT levels

The distribution of genotypes for *OXTR* (rs53576) in the participant group was as follows: 22 GG (14.8%), 65 AG (43.6%), and 62 AA (41.6%) genotypes. Prior to statistical analysis, we confirmed that no significant deviation from the Hardy–Weinberg equilibrium was observed in participants [*χ* ^2^(1) = 0.53, *p* = 0.47]. This distribution was similar to those observed in other studies involving Asian participants^27^.

**Figure 4:**
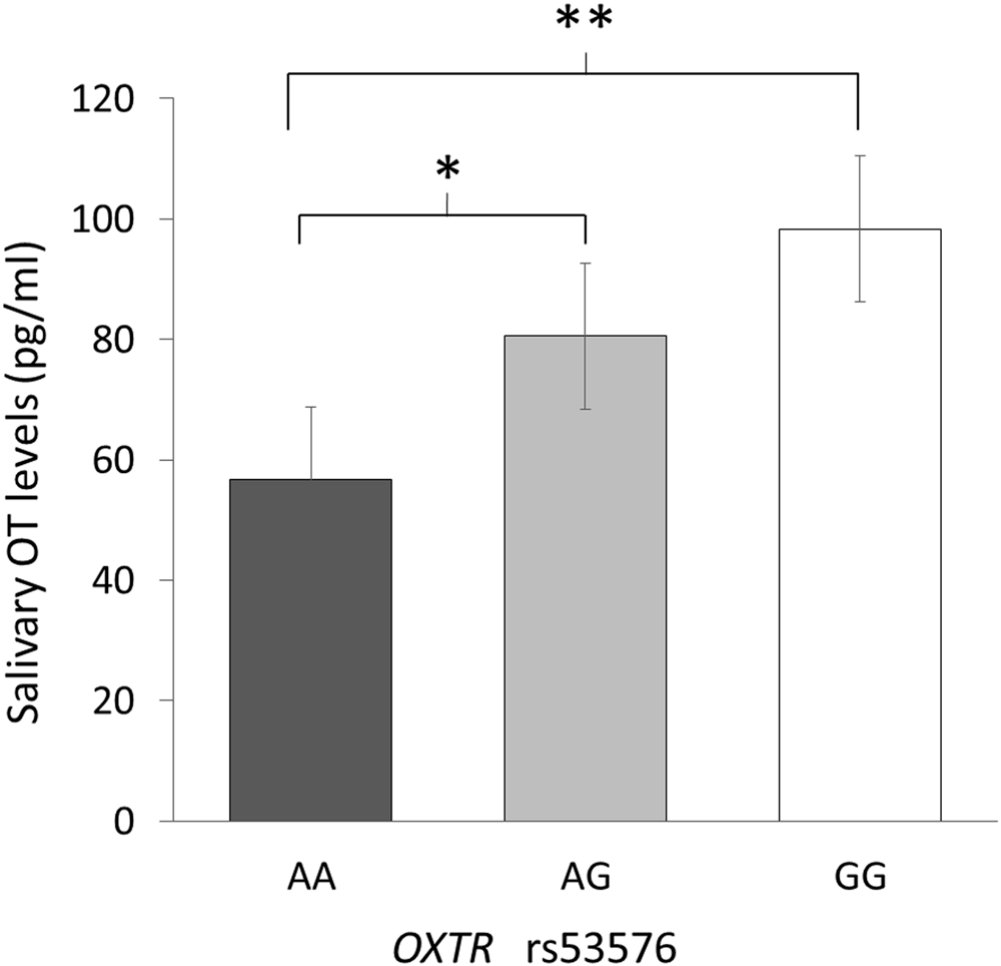
Relationship between *OXTR* polymorphisms and salivary oxytocin (OT) levels. The vertical axis indicates salivary OT levels (pg/ml). The horizontal axis indicates the *OXTR* genotypes. *p < 0.05; **p < 0.01.

To examine the effect of the *OXTR* rs53576 genotype on salivary OT levels, a one-way analysis of variance (ANOVA) was performed using participants’ genotypes as a between-participants factor (Fig. 4). The analyses revealed a significant main effect of genotype [*F* (2,146) = 4.461, *p* = 0.01]. *Post hoc* analysis using Shaffer’s modified sequentially rejective Bonferroni procedure revealed that individuals with the AA homozygote genotype (mean = 56.72, SD = 45.17) had lower salivary OT levels than individuals with a G allele (AG: mean = 80.54, SD = 68.08; GG: mean = 98.37, SD = 77.86, respectively). This result suggests that salivary OT levels in young children are modulated by the *OXTR* rs53576 polymorphism, with AA genotype carriers exhibiting significantly lower salivary OT levels than G allele carriers.

### Effects of age and *OXTR* polymorphisms on gaze fixation for social cues

**Figure 5:**
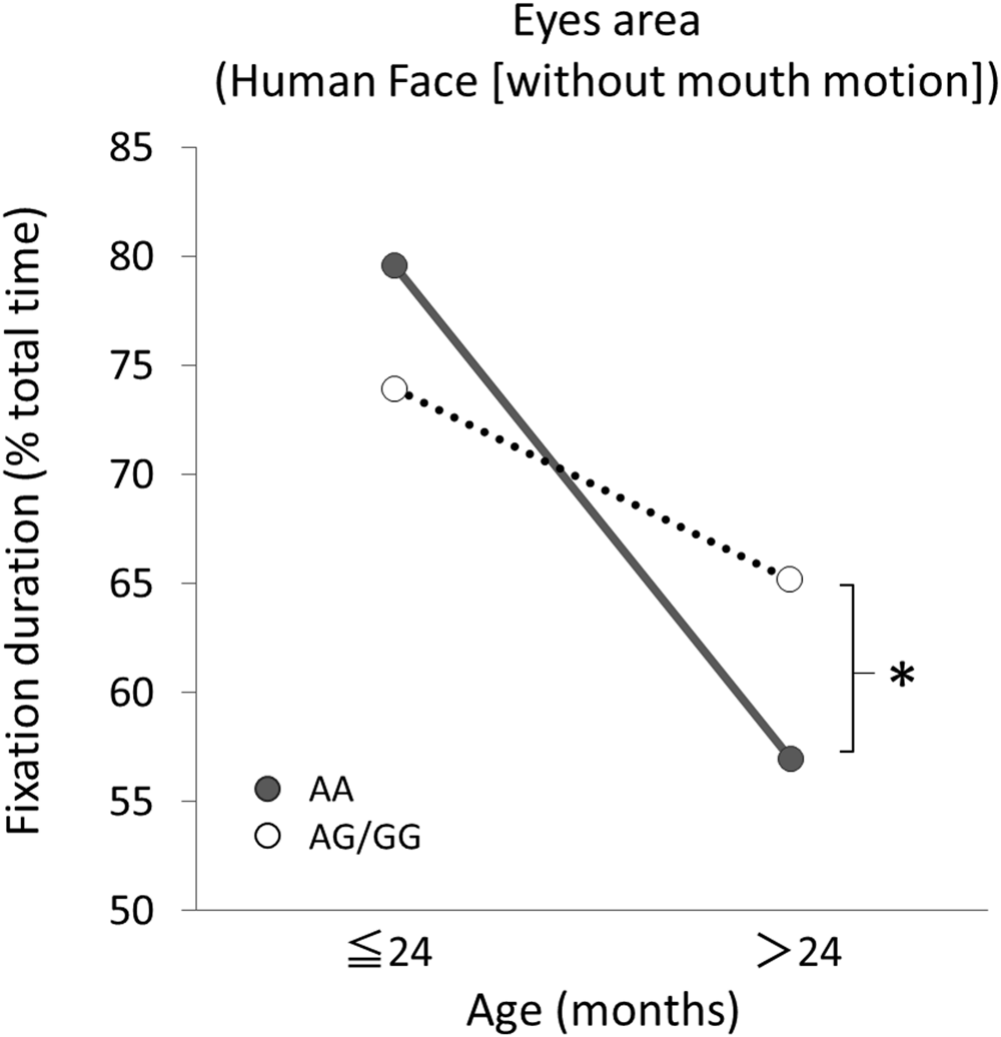
Interaction between genotype and age in visual attention to the eyes as social cues. Solid line is the *OXTR* rs53576 AA group (n = 62); dotted line is the *OXTR* G-carrier group (n = 87). *p < 0.05.

Human cognitive development, including visual attention, appears to change from the sensorimotor period to the preoperational period, which occurs at the age of approximately 24 months^28, 29^. Research has also indicated that the role of gaze fixation may change significantly over the second year of life^30^. Therefore, the data were analysed to ascertain whether an interaction effect between age and *OXTR* polymorphisms existed in relation to visual attention for social cues. A two-way ANOVA was conducted, including age below 24 months and from 24 months and older, *OXTR* polymorphisms (AA genotype or G allele), and the fixation duration of each AOI in the face. This analysis indicated that only the fixation duration for the eye area of the face (without mouth motion) was associated with a significant interaction between age and *OXTR* genotype from 24 months onwards [*F* (1,145) = 5.53, *p* = 0.02]. No main was observed for either genotype or age (Fig. 5).

There were no significant associations in any other AOIs of the face. Upon further examination of the interactions, *post hoc* analysis revealed a simple main effect of genotype, indicating that children over 24 months of age with the AA genotype exhibited a significantly decreased fixation duration on the eye area compared to G allele carriers [*F* (1,145) = 4.07, *p* = 0.04]. No simple main effect of genotype was observed in children under 24 months of age [*F* (1,145) = 1.75, *p* = 0.19].

## Discussion

We investigated the developmental relationships between gaze fixation for social cues and OT levels in infants and young children between the ages of 6 and 90 months. Our analysis yielded four main findings. First, we observed a parallel decrease in both attention to social cues (e.g. eyes, people, or finger pointing) and salivary OT levels with age during infancy and early childhood, as well as an increase in attention for non-social cues (e.g. geometry or non-pointed-at objects). Second, we observed significant associations between attention to the eye and mouth areas of the face and salivary OT levels. Third, we also revealed that children with the *OXTR* AA homozygous genotype exhibited lower salivary OT levels than those with G alleles. Finally, visual attention on the eye area was associated with both *OXTR* polymorphisms and age.

Several previous studies have suggested that OT exerts a positive effect on attention for social cues, including the eye region^1–3, 7^. Further, impairments in social functioning, such as those associated with ASD, have been related to dysfunction of the oxytocinergic system^31–33^. Previous eye-tracking studies have revealed that children with atypical development (including those with ASD) exhibit lower or altered visual attention to social cues^24^. Although we have examined such associations in our previous studies^22, 34, 35^, to the best of our knowledge, the present study is the first to clarify the association between developmental changes in the pattern of visual attention to the eyes as social cues and OT levels during young childhood.

Our results offer new information, indicating that attention to most types of social cues in infants and young children decrease with age, whereas attention to non-social cues increases with age. Additionally, the trajectories of age-related changes in social attention may be dependent on the types of social cues used (e.g., face, people, or finger pointing). Although a handful of studies regarding developmental changes in visual attention for social cues have been published^22, 34, 35^, the decrease observed in the present study is partially consistent with the findings of a previous study, which reported that gaze fixation increases with age in infants^35^.

Our results also indicate that salivary OT levels decrease with age. Although only a handful of studies have investigated the effects of age on the human oxytocin system^36^, age-related decreases in OT levels have been observed in infant rhesus macaques^37^. Furthermore, one previous study revealed that mother-infant interactions are positively associated with endogenous OT levels in human mothers and their children^14^. These findings suggest that the observed decrease in OT levels may reflect a reduction in direct interaction between children and their mothers, such as physical contact or being together, with aging (e.g., infants spend more time interacting with their mothers than toddlers or younger children).

The most curious finding of the present study was the significant parallel decrease between salivary OT levels and the duration of gaze fixation on the eye and mouth areas. When controlled for age, weak positive correlations between OT levels and gaze fixation on the eye area (with mouth motion) were observed. Research on human communication has suggested that OT facilitates the ability to infer the mental state of others from the eye region in adults^2, 3, 32^ and during parent-infant interactions^14, 38^. Therefore, we speculate that higher OT during young childhood plays a significant role in executing efficient social interactions between the mother and child via eye contact.

In the present study, we observed that children with the AA homozygous genotype had lower salivary OT levels than those with G alleles. However, previous studies have suggested no association between peripheral OT levels and *OXTR* rs53576 polymorphisms in either children or adults^39, 40^. The inconsistency between our findings and those of previous studies may be attributable in part to differences in OT levels or *OXTR* genotype frequencies based on demographic characteristics of the population, such as age or ethnicity^39, 40^.

As previously reported^17, 41^, the *OXTR* rs53576 AA homozygous genotype, associated with decreased sociality, was associated with lower OT levels in the present study. Further, *OXTR* rs53576 AA homozygotes demonstrated lower fixation duration on the eye area compared to G-allele carriers among children aged 24 months and older. Only one previous study has reported an association between visual attention to the eyes and a gene polymorphism related to oxytocin (CD38) in young children^42^. Together, these studies suggest that social eye cues can be manipulated by genetic factors as well as endogenous levels of oxytocin. Although our findings are largely consistent with those of the previous study, the interaction was apparent only after 24 months of age in the current study. One possible explanation for this is the establishment of multidimensional processes of social synchrony with other social bonds, such as those with peers, after 24 months of age^28, 43^. With continued investigation, this line of research may aid in the development of critical early screening tools, allowing for the identification of infants at high risk for developing social impairments based on *OXTR* polymorphisms and low OT levels in early childhood.

Several limitations of the present study should be noted and taken into consideration in future studies. First, this study included a relatively small sample of participants and utilised a cross-sectional design that precluded the identification of causal links between the development of social attention and OT systems. Longitudinal studies utilizing larger sample sizes are required in order to more fully elucidate the association between development of the OT system and visual attention for social cues. Second, although we used unextracted samples to measure OT levels for two major reasons in the current study (see Methods), recent studies have reported inconsistent findings regarding the relationship between extracted and unextracted samples for the measurement of peripheral OT levels^44, 45^. Further research is also required to more fully elucidate the neurobiological determinants of salivary OT levels. In addition, we were unable to examine the effect of TP levels on OT levels^46^ in all participants, as the volume of salivary samples was insufficient in some participants. Although we observed an association between OT levels and age regardless of TP levels in a subset of participants, future studies should verify this finding by measuring salivary OT levels for all samples obtained. Third, we did not exclude developmental disabilities from our analyses or assess socioeconomic status and individual parenting style. Hence, we cannot conclude whether our approach (i.e., the combination of eye-tracking using the Gazefinder, measurement of salivary OT levels, and assessment of *OXTR* polymorphisms) is useful for early screening in infants. Fourth, the non-invasive nature of the Gazefinder, which provides new insight into social function, may be of particular advantage in the examination of children, allowing clinicians to monitor patterns of gaze fixation when screening for ASD. However, our findings regarding social functioning in the present study do not necessarily imply diagnostic importance, as the data are descriptive of physical and functional condition. Finally, other polymorphisms or gene-gene interactions were not taken into consideration^47, 48^. As recently suggested, ethnic differences in *OXTR* genetic effects and certain forms of culture-specific relational norms may also be important^27^. Therefore, future studies should also include *OXTR* polymorphism data in the path analysis.

In conclusion, our results demonstrate that both attention for social cues (eyes, people, pointing area) and salivary OT levels are negatively associated with age, whereas attention for non-social cues (mouth, geometry, non-pointed area) is positively associated with age. Furthermore, OT levels are positively associated with visual attention for human faces, especially for the eye area. These results suggest that OT is involved in visual attention for eyes but that such associations are largely dependent on age. In addition, OT levels are modulated by the *OXTR* rs53576 polymorphism in children after 24 months of age, and the AA genotype is significantly associated with decreased fixation time on the eye area, relative to that in children with G alleles. These results suggest that the development of attention for eyes is modulated by the *OXTR* polymorphism, which exerts an effect on OT levels. Thus, combining this experimental paradigm with neurophysiological indicators of brain activity (such as imaging techniques) should prove fruitful in further elucidating mechanisms underlying visual attention for social cues.

## Methods

### Participants

One hundred and forty-nine infants and children (76 boys, 73 girls; mean age: 33.6 ± 24.3 months; age range: 5–90 months) and their mothers, who were recruited from the local community via advertisements, participated in the present study. The ethnicity of all participants was Japanese. We did not perform psychodevelopmental evaluations of the participants. However, if the presence of developmental problems was suspected in candidates for this study, we excluded them from our recruitment. No participants had any history of any form of epilepsy or abuse, head injury, or foetal drug exposure that might have influenced brain development. We did not measure socioeconomic status.

The study protocol was approved by the Ethics Committee of the University of Fukui (Assurance no. 20140142) and was conducted in accordance with the Declaration of Helsinki and the Ethical Guidelines for Clinical Studies of the Ministry of Health, Labour, and Welfare of Japan. The parent(s) of all participants provided written informed assent and consent for participation in this study.

### Measurement of gaze patterns

We measured each participant’s gaze pattern using Gazefinder (JVC KENWOOD Corporation, Kanagawa, Japan), an all-in-one eye-tracking system for responses to social cues by visual stimuli^25, 49^. The Gazefinder used infrared light sources and cameras that were integrated into a 19-inch-thin film transistor monitor (1280 × 1024 pixels). Using corneal reflection techniques, the Gazefinder records the X and Y coordinates of each child’s eye position at a frequency of 50 Hz (i.e., 3000 data collections/minute). Stimuli presented by the Gazefinder consisted of short movies including four categories of social cues, which were (a) human faces (with or without mouth motion), (b) people and geometric patterns, (c) biological motion of a human, and (d) objects with or without finger pointing. Additionally, the category (a) of human faces was divided into two subcategories. One subcategory is included stimuli without mouth motion, such as staring with silence, while the other included stimuli with mouth motion, such as talking (e.g., “Hello” or “What’s your name?”).

Moreover, two areas-of-interest (AOI-1 and AOI-2) were set within each stimulus. AOI-1 was a target area considered to represent higher social cues [“eyes” in (a), “people” in (b), “upright figure” in (c), and “object with pointing” in (d)]. AOI-2 was a control area considered to represent lower or non-social cues [“mouth” in (a), “geometry” in (b), “inverted figure” in (c), and “objects without pointing” in (d)] (For more details, refer to previous studies^25, 49^).

### Procedure and stimuli

The experiments were conducted at a research laboratory located in the Research Center for Child Mental Development at the University of Fukui, Japan. Children were seated (on a parent’s lap when younger than 24 months old, and mothers were instructed not to assist their children) 40 cm in front of the eye-tracking monitor. To obtain calibration information, the participants were initially asked to look at images of an animated animal that appeared in one of five locations on the screen. If the calibration quality was poor for any of these points, the calibration process was repeated. Before the task, children were instructed that pictures of faces, people, and objects were to be shown on the computer screen and that they should look at them without looking away for as long as possible. Stimulus movies were displayed in a definitive order^49^. Before each trial, an attention-getting animation with a voice saying “Hey! Look!” was presented in the centre of the screen to reorient the child’s attention to the stimuli.

### Measurement of salivary OT levels

Saliva samples were collected using Salivettes® (Sarstedt, Rommelsdorft, Germany) after the children had viewed the stimulus movies. Parents were asked to put a roll of cotton in their child’s mouth and instruct their child to chew for 1 minute until it was saturated with saliva. Two cotton samples were collected by repeating this process. Saliva samples were frozen and stored at −80 °C. Before the assay, saliva samples were lyophilized overnight and kept at −20 °C to concentrate them two to four times. The dry samples were reconstituted in the assay buffer immediately before analysis using a commercial OT enzyme immunoassay kit (Enzo Life Sciences, Inc., NY, USA). These protocols were consistent with those of an earlier study on adults^50–52^, as well as with those of a study conducted with 3-year-old children^14^ and of our previous study^25^.

In the current study, we used unextracted salivary samples for the following reasons. First, as analysis of unextracted salivary samples revealed a more than 80% recovery rate as well as acceptable matrix interference, we considered extraction unnecessary. Second, previous reports have suggested that the extraction process may remove the majority of peripheral oxytocin, including oxytocin that is bound to other molecules in the blood, such as albumin^53^. The kit utilised in the present study is exclusive to OT, eliminating the influence of other peptides such as arginine, vasopressin, and somatostatin. Each sample was examined in duplicate, and concentrations were calculated using the SpectraMax® (Molecular Device, Sunnyvale, California) micro plate reader, according to relevant standard curves. Average intra-and inter-assay coefficients of variation (CV) were 6.5% and 9.4%, respectively.

We also measured salivary protein levels using a commercial protein assay kit (Thermo Fisher Scientific Inc., Waltham, MA, USA) to investigate the effect of TP on age-associated changes in OT levels (Supplementary Information). However, this analysis was performed only for a subset of participants (n = 54), as the volume of saliva samples was insufficient for some infants and children. We also measured salivary blood contamination levels^54^ using a commercial EIA kit (Salimetrics Inc., State College, PA, USA) in a subset of participants. The results of this analysis revealed that the level of blood contamination was within the acceptable range for the samples (M = 0.43 ± 0.3 mg/dl).

### Genotyping

Genomic DNA was extracted from the buccal mucosa cells using a standard phenol-chloroform method using the QIAamp DNA Micro Kit (QIAGEN, Tokyo, Japan). We targeted the *OXTR* SNP (rs53576), which was selected due to its significant association with “sociality” in human behaviour in a previous meta-analysis^17^. SNPs were genotyped using TaqMan genotyping assays (Applied Biosystems, Foster City, CA, USA) and the standard protocols provided by the manufacturer. All samples were genotyped via real-time polymerase chain reaction (PCR) analysis using the StepOnePlus System (Applied Biosystems, Foster City, CA, USA). Reactions were performed in a 10 μL volume, containing 9 ng genomic DNA, 0.25 μL of Tris-EDTA buffer, 0.25 μL of each TaqMan probe, and 5 μL TaqMan PCR Master Mix. The PCR cycling conditions consisted of one 10-minute cycle at 95 °C, followed by 60 cycles at 95 °C for 30 seconds and 60 °C for 30 seconds. In each amplification, 4.5 μL HPLC water plus Master Mix was used as a negative PCR control. Genotype discrimination was then conducted using StepOnePlus System software (version 3.0.1.).

### Statistical analysis

We computed the percentage of time spent with gaze fixated on the two AOIs, as well as on other areas on the screen, and analysed them as dependent variables. First, we calculated the correlation coefficients to examine the associations between the percentage of fixation time for each AOI, OT levels, and age. Next, we conducted an analysis of variance (ANOVA) to compare OT levels among the rs53576 polymorphisms. Finally, an ANOVA was used to assess the effects of both genotype and age on social attention. The significance level was set to *p* < 0.05. Statistical analysis was conducted using R software (version 3.2.0. for Windows, R), and ANOVAs were executed using “anovakun” in R software (version 4.8.0.)^55^.

## Electronic supplementary material

Supplementary Information
