# Supplementary material for: Developmental changes in social attention and oxytocin levels in infants and children
Source: Sci Rep. 2017 May 31;7:2540. doi: 10.1038/s41598-017-02368-x (PMC5451468; doi:10.1038/s41598-017-02368-x)

Supplementary Information

# Developmental changes in social attention and oxytocin levels in infants and children

Minaho Nishizato, Takashi X. Fujisawa, Hirotaka Kosaka, Akemi Tomoda*

## Effects of salivary total protein levels on age-associated changes in salivary oxytocin levels

Salivary secretion is necessary for oral health, fulfilling mechanical cleansing and protective functions thorough a number of physiological and biochemical mechanisms. Salivary composition varies throughout infancy, reflecting the development of the salivary glands and leading to variations in protein concentration in the saliva of infants and children.[1,2] Previous reports have indicated that the total protein (TP) levels in saliva change with age[3], suggesting that oxytocin (OT) levels in saliva may depend on salivary TP levels. Thus, we investigated the association among salivary OT levels, salivary TP levels, and age.

* Corresponding author: Tel: +81-776-61-8677, Fax: +81-776-61-8678, E-mail: atomoda@u-fukui.ac.jp

We also measured salivary TP levels in a subset of participants (n = 54; 31 boys, 23 girls; mean age: 43.6 ± 23.6 months; age range: 5-87 months) to investigate the effect of TP levels on age-associated changes in OT levels. (The analysis could not be performed for all participants, as it was difficult to obtain sufficient volumes of saliva from some infants and children.) Saliva samples were collected using Salivettes® (Sarstedt, Rommelsdorft, Germany), following which they were were frozen and stored at −80°C. The samples were assayed using a commercial protein assay kit (Pierce™ 660nm Protain Assay, Thermo Fisher Scientific Inc., Waltham, MA, USA). Each sample was examined in duplicate, and concentrations were calculated using the SpectraMax® (Molecular Device, Sunnyvale, California, USA) micro plate reader, according to relevant standard curves. Average intra-and inter-assay coefficients of variation (CV) were 3.3% and 5.8%, respectively.

In this subset of participants, we observed a significant negative correlation between salivary OT levels and age ($r$ = -0.423, $p$ = 0.001) (Figure S1a), although there was no significant correlation between salivary TP levels and age ($r$ = 0.211, $p$ = 0.126) (Figure S1b). We then calculated partial correlation coefficients between salivary OT levels and age, controlling for TP levels. Our results revealed a significant negative partial correlation between OT levels and age ($r$ = -0.390, $p$ = 0.004). Similarly, a

negative correlation was also observed between salivary OT levels normalized by salivary TP levels and age ($r$ = -0.328, $p$ = 0.015). These results suggest that salivary OT levels decreased with age regardless of TP levels in each participant.

Thus, our findings regarding salivary TP levels during infancy and childhood are consistent with those of previous reports, which have reported either an increase or no change in the amount of TP during childhood[1,4]. On the other hand, only a handful of studies have investigated the effects of age on the human oxytocin system[5]. Although there is no direct evidence of decreasing OT levels with age in human studies, age-related decreases in OT levels have been observed in infant rhesus macaques[6]. Additionally, a previous human study revealed that mother-infant interactions are positively associated with endogenous OT levels in both mothers and their children[7]. These findings suggest that the decreases in OT levels observed in the present study may reflect a reduction in direct interaction between children and their mothers, such as physical contact or being together, with age (e.g., infants spend more time interacting with their mothers than toddlers or younger children). Taken together, these findings also support the notion that salivary OT levels decrease with age regardless of salivary TP levels during infancy and childhood.

**References**

1 Dezan, C. C., Nicolau, J., Souza, D. N. & Walter, L. R. F. Flow rate, amylase activity, and protein and sialic acid concentrations of saliva from children aged 18, 30 and 42 months attending a baby clinic. *Arch Oral Biol* **47**, 423-427, doi:http://dx.doi.org/10.1016/S0003-9969(02)00032-8 (2002).

2 Sivakumar, T., Hand, A. R. & Mednieks, M. Secretory proteins in the saliva of children. *J Oral Sci* **51**, 573-580 (2009).

3 Feldman, R., Gordon, I. & Zagoory-Sharon, O. The cross-generation transmission of oxytocin in humans. *Horm Behav* **58**, 669-676, doi:10.1016/j.yhbeh.2010.06.005 (2010).

4 Wu, K. P. *et al*. Relationship between unstimulated salivary flow rate and saliva composition of healthy children in Taiwan. *Chang Gung Medical Journal* **31**, 281-286 (2008).

5 Huffmeijer, R., van Ijzendoorn, M. H. & Bakermans-Kranenburg, M. J. Ageing and oxytocin: a call for extending human oxytocin research to ageing populations--a mini-review. *Gerontology* **59**, 32-39, doi:10.1159/000341333 (2013).

6 Parker, K. J., Hoffman, C. L., Hyde, S. A., Cummings, C. S. & Maestripieri, D. Effects of age on cerebrospinal fluid oxytocin levels in free-ranging adult female and infant rhesus macaques. *Behav Neurosci* **124**, 428-433, doi:10.1037/a0019576 (2010).

7 Feldman, R., Gordon, I., Influs, M., Gutbir, T. & Ebstein, R. P. Parental oxytocin and early caregiving jointly shape children's oxytocin response and social reciprocity. *Neuropsychopharmacology* **38**, 1154-1162, doi:10.1038/npp.2013.22 (2013).

**Figure S1.** (a) Association between age and salivary oxytocin levels. The vertical axis indicates salivary oxytocin (OT) levels (pg/ml), whereas the horizontal axis indicates age. (b) Relationship between age and salivary total protein (TP) levels. The vertical axis indicates salivary oxytocin levels (mg/ml), whereas the horizontal axis indicates age. ***$p$ = 0.001.

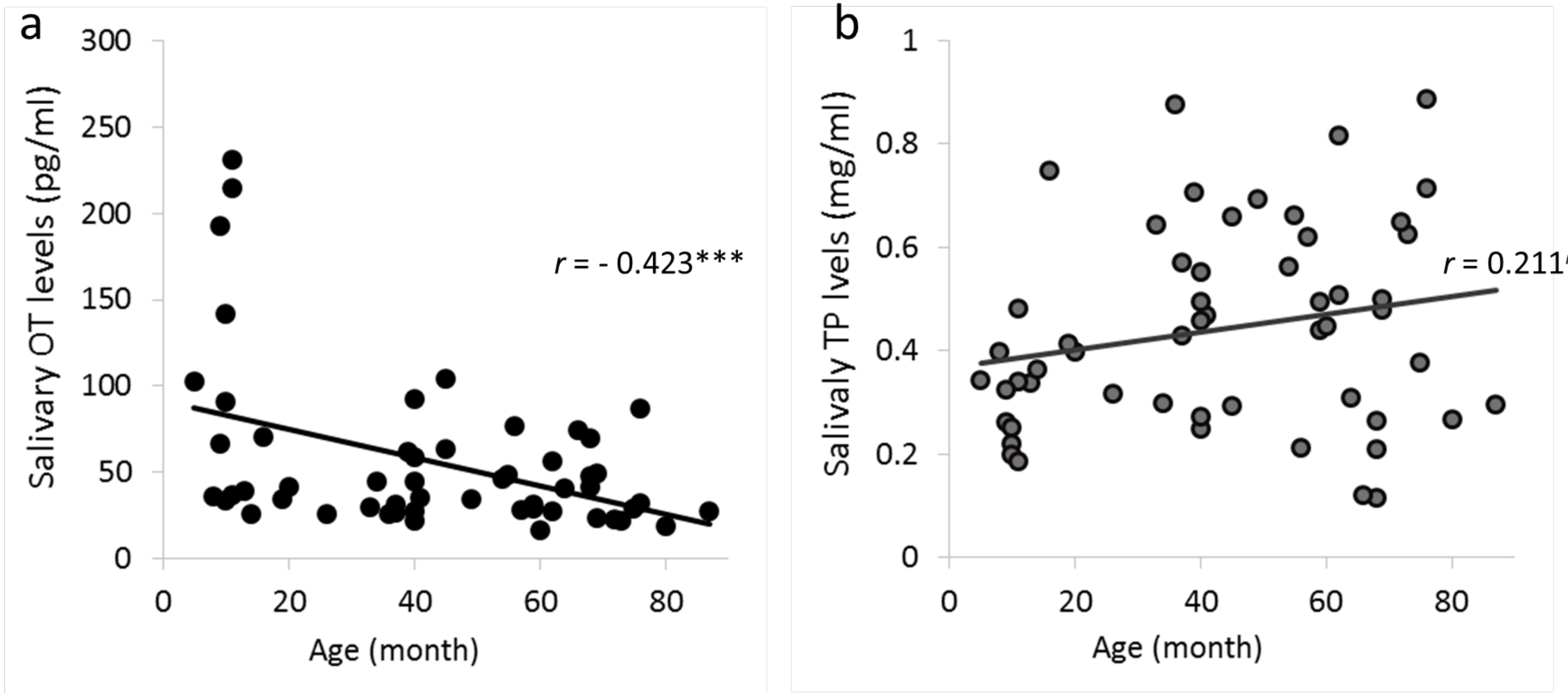

Supplement: Supplementary file 1 — Supplementary Information [file 41598_2017_2368_MOESM1_ESM.pdf]
